# Officers' Food and the Regulation of Meals

**Published:** 1886-10-16

**Authors:** 


### The Editor's Letter-Box.

[Our Correspondents are reminded that prolixity is a great bar to publication, and that brevity of style and conciseness of statement greatly facilitate early publication.]

### OFFICERS' FOOD AND THE REGULATION OF MEALS.

"Hospital" writes:—"I cannot coincide with the views expressed by 'A Hospital Manager and Trustee.' I am the senior resident officer of one of the largest metropolitan hospitals, where we have a mess of nine, and it is very rarely that there is any grumbling or complaint. The same state of things exists at the nurses' table, from which, I am glad to say, I seldom hear of any dissatisfaction. Our dinner consists of soup or fish, joint, and sweets on five days of the week, and on the other two the soup or fish is omitted. Table-beer is served to the officers at luncheon and dinner, but the consumption is not nearly so great now as it was a decade ago when I first entered hospital life. Breakfast is on the table from 8·30 to 9·30, luncheon from 12·30 to 2, and dinner at 6·30. The best remedy for irregularities is a little tact in administration." Hospital.

